# Radiotherapy and the Tumor Stroma: The Importance of Dose and Fractionation

**DOI:** 10.3389/fonc.2014.00001

**Published:** 2014-01-21

**Authors:** Turid Hellevik, Iñigo Martinez-Zubiaurre

**Affiliations:** ^1^Department of Oncology, University Hospital of Northern-Norway, Tromsø, Norway; ^2^Translational Cancer Research Group, Department of Clinical Medicine, University of Tromsø, Tromsø, Norway

**Keywords:** stereotactic ablative radiotherapy, tumor microenvironment, hypoxia, angiogenesis, cancer-immunity, cancer-associated fibroblasts

## Abstract

Ionizing radiation is a non-specific but highly effective way to kill malignant cells. However, tumor recurrence sustained by a minor fraction of surviving tumor cells is a commonplace phenomenon caused by the activation of both cancer cell intrinsic resistance mechanisms, and also extrinsic intermediaries of therapy resistance, represented by non-malignant cells and structural components of the tumor stroma. The improved accuracy offered by advanced radiotherapy (RT)-technology permits reduced volume of healthy tissue in the irradiated field, and has been triggering an increase in the prescription of high-dose oligo-fractionated regimens in the clinics. Given the remarkable clinical success of high-dose RT and the current therapeutic shift occurring in the field, in this review we revise the existing knowledge on the effects that different radiation regimens exert on the different compartments of the tumor microenvironment, and highlight the importance of anti-tumor immunity and other tumor cell extrinsic mechanisms influencing therapeutic responses to high-dose radiation.

## Introduction

Radiotherapy (RT) is a long-standing pillar in both curative and palliative cancer treatment, and about 50% of all cancer patients are receiving RT some time during their disease (1). However, despite the proved efficacy of RT, many treated cancer patients suffer from locally recurrent disease and/or metastatic spread of tumor growth (2). In the clinics, implementation of advanced imaging, computer technology together with technical perfection in radiation therapy planning and performance, has revolutionized the field (3). Consequently, a different therapeutic strategy commonly referred to as stereotactic ablative radiotherapy (SART), stereotactic body radiotherapy (SBRT), or stereotactic radiosurgery (SRS) is now gaining terrain in the clinics for the treatment of cancers with difficult cures such as lung, liver, pancreas, or brain (4, 5).

In line with the notion that solid tumors are complex and heterotypic tissues (6, 7), it is currently recognized that cancer cell responses to therapeutic treatments are greatly influenced by their microenvironment (8–10). This includes the physical contact of tumor cells with structural elements such as ECM via integrins, associations with resident and temporary host cells such as inflammatory cells, vascular cells, fibroblasts, and circulating progenitor cells, and interactions with miscellaneous diffusible molecules of autocrine, paracrine, and endocrine origin that influence cell behavior (2, 11). In RT, the nature of both external and internal RT is unspecific in the sense that it does not exclusively target neoplastic cells but necessarily affects all tumor-associated cells. Accordingly, secondary effects inflicted by RT on benign tumor constituents markedly guide the therapeutic outcomes post-treatment. Intriguingly, the radiation-induced changes in different stromal constituents of tumors have been demonstrated to depend on physical parameters such as total radiation dose, fraction-size, fraction intervals, or number of fractions. In view of that, recent reports have underscored the existence of threshold doses (and regimens) that are able to switch on different damage programs that profoundly affect responses to therapy. For instance, it has been suggested that local high-dose irradiation exerts more potent immune-responses than standard low-dose radiation, therefore promoting the eradication of cancer cells that escape the radiation-induced death (12). Studies with experimental tumors show that irradiation with doses higher than 10 Gy in a single-fractioned or up to 60 Gy in an oligo-fractionated manner causes severe vascular damages leading to strong alterations of the tumor microenvironment and indirect death of tumor cells (13). Furthermore, exposure of tumor fibroblasts to high radiation doses but not to small doses induces permanent DNA-damage responses and irreversible cellular senescence, which in turn might influence therapeutic upshots by the altered release of cytokines, chemokines, and growth factors (14). Given the acknowledged role played by the tumor microenvironment on guiding responses to therapy and the growing interest on using hypofractionated high-dose RT on cancers with poor prognosis, here we have aimed at presenting an overview on existing knowledge on how the different cellular compartments in tumors respond to different radiation schemes, and their potential impact on treatment outcomes. Summarized key literature is presented in Table 1.

**Table 1:** **Responses of the various cellular components of the tumor stroma to diverse radiation regimens**.

Cell type	Tumor type	Experimental model	Radiation schemes	Effects	Reference
Endothelial cells	AVM (no tumor)	Human specimens	15–50 Gy × 1	Damage to EC is the earliest change after irradiation; >75% size reduction in AVM (Arterio-Venous Malformation)	(70)
	Brain metastasis	*In silico*	20 Gy × 1	Occlusion of ≥99% of vessels within 1 year post-RT Vascular effect calculated to contribute by 19–33% of overall effect	(59)
	Sarcoma Melanoma	*In vivo In vitro*	10 Gy × 1	*In vitro:* EC apoptosis above 10 Gy *In vivo*: apoptosis induced in ECs above 15 Gy (local RT)	(61)
	–	*In vivo*	(a) 8–13 Gy	(a) Threshold-value for induction of EC-apoptosis, 1–6 h post-WBR	(63)
			(b) >17–18 Gy	(b) Endothelial-independent GI damage activated; 8–24 h post-WBR	
	Sarcoma Melanoma	Xenografts in *asmase*^+/+^ or ^−/−^mice	13.5 Gy × 1 WBR	Anti-VEGFR2 given (0.5–2 h) before RT upregulates ceramide levels, resulting in enhanced apoptotic fraction of ECs. Anti-angiogenic effect fails without elevated ceramide levels	(64)
	–	Xenograft and human specimens	(a) <5 Gy	(a) Tumor vasculature preserved or improved	(13)
			(b) 5–10 Gy	(b) Mild vascular damage	
			(c) <10 Gy	(c) Severe vascular damage, indirect tumor cell death	
Immune cells	Sarcoma	Mice	10 Gy × 3	Complete tumor regression by combining DC-immunotherapy and high-dose RT; no effect as single therapies	(160)
	Melanoma Sarcoma	Mice	8.5 Gy × 5	Local and systemic anti-tumor response by combining DC administration and local oligo-fractionated RT	(161)
	Melanoma	Mice	15 Gy × 1	Increased accumulation of effector CD8^+^ T-cells upon local RT	(131)
			3 Gy × 5	Stronger immune-responses by single high-dose RT	
	Melanoma	*In vitro*	(1, 4, 10, 25) Gy × 1	A marked increase in cell-surface MHC class-I expression observed at higher doses (10–25 Gy) over a period of 3 days	(108)
	Colon cancer	Mice/humans	10 Gy × 1	Danger signals released by dying cells after RT as key events for mounting adaptive immune-responses	(117)
	Breast cancer				
	Sarcoma				
	Melanoma	Mice	(15–25) Gy × 1	Local and systemic anti-tumor effects after ablative RT depends on CD8^+^ T-cell activation	(12)
	Lung				
	Melanoma	Mice	25 Gy × 1	Local ablative RT trigger intratumoral production of IFN-β, resulting in enhanced cross-priming ability of DCs and tumor regression	(134)
	Colon	Mice	10 Gy × 1	High-dose RT elicits tumor-specific immunity by activation of tumor-associated DCs and CD8^+^ T-cells, but not via CD4^+^ or macrophages	(135)
	Lung				
	Melanoma				
Normal fibroblasts	Normal fibroblasts	*In vitro*	(0.5, 2, 5, 15, 50) Gy × 1	Normal fibroblasts (NFs) survive a radiation dose of 50 Gy Human NFs exposed to 15 Gy resulted in the highest number of	(140)
				up- and down-regulated genes, peaking at 24 and 48 h post-IR	
	Squamous cell carcinoma	*In vitro*	12 Gy 24 Gy	Irradiated NFs promoted growth and invasion of non-irradiated SCC tumor cells. 12 Gy induced the greatest invasion. TGF-β expressed only by irradiated fibroblasts	(162)
	Skin fibroblasts	*In vitro*	0.5 Gy × 1 10 Gy × 1	Persistent DNA-damage signaling only at 10 Gy. High-dose induction of irreversible cell senescence and initiation of cytokine response	(147)
	Lung	Primary lung fibroblasts	(5, 15, 20, 25) Gy × 1	Cytokine production by NFs exposed to escalating RT doses. RT doses above 15 Gy triggers enhanced expression of TGF-β	(138)
				IL-6, IL-8, and MCP-1 expression by NF unchanged post-RT	
	Lung	*In vitro In vivo*	4 Gy × 12	Human NFs become senescent after an accumulative dose of 50 Gy, and turn pro-tumorigenic by increased expression of MMP1	(145)
Cancer-associated fibroblasts	Pancreatic cancer	Co-cultures: CAFs + adeno-carcinoma cells	5 Gy 10 Gy	Enhanced invasiveness of pancreatic cancer cells co-cultured with irradiated CAFs, blocked by antagonist to HGF. Secreted HGF-levels unchanged after high-dose RT; bFGF-levels enhanced	(157)
	Pancreatic cancer	*In vitro In vivo*	100 Gy × 1	Conditioned medium from human pancreatic stellate cells protects pancreatic tumor cells from radiation-induced apoptosis	(163)
	Breast cancer	Primary CAF cultures	30 Gy × 1	Breast CAFs and normal fibroblasts (NF) exhibit high radio-resistance. CAFs proliferate faster than NFs, and express higher levels of the tumor protecting factor Survivin	(144)
	Pancreatic cancer	Tumor-derived primary cells and cell lines	3.5 Gy × 3	Pancreatic stellate cells promote radioprotection of cancer cells in a β1-integrin dependent manner, and stimulate proliferation of pancreatic cancer cells in direct co-culture	(164)
	Non-small cell lung cancer (NSCLC)	*In vitro* human primary CAFs	(2, 6, 12, 18) Gy × 1	>12 Gy permanent DDR and induction of cellular senescence	(146)
				At ablative RT doses: reduction of proliferative and migratory abilities. Induction of cell surface focal contacts	
	NSCLC	*In vitro* human primary CAFs	18 Gy × 1	Secretome-analysis after ablative RT: reduced expression of angiogenic factors SDF-1, Angiopoietin-1, TSP-1; elevated levels of bFGF; unchanged levels of HGF, IL-6, IL-8, Il-1β, and TNF-α	(14)

## The Evolution of Radiation Therapy and Treatment Regimens

Landmark observations about 80 years ago suggested that normal tissues could be spared if the radiation dose was delivered in fractions over several days (15). Since then, curative RT has typically been delivered as multi-fractionated rather than single-dose regimens, with daily doses of 2 Gy (5×/week), to an accumulative dose of 50–70 Gy. Big leaps within RT-related imaging and technology have led to enhanced accuracy, thus reducing the need of including large safety margins (of healthy normal tissue) in the treatment field. Hence, on the condition of rigorous quality assurances (16, 17), high-doses of ionizing radiation (IR) may now be delivered safely to patients.

The first attempts to deliver ablative or high-dose RT to patients in a single session derive from 1951, when neurosurgeon Leksell constructed an immobilizing stereotactic head-frame for delivery of highly focused IR with extreme precision to small volumes of non-malignant lesions of the brain (18). Later on, modified versions of this non-invasive technique, named “SRS,” have been applied to small primary tumors and single metastases of the brain (19, 20). Single fractions of 20–30 Gy are typical in the context of SRS, resulting in high rates of local tumor control, preservation of cerebral functions, and option of repeated treatment (21). Recently, oligo-fractionated SRS has proven beneficial also for brain tumors that are large (>3 cm) (22, 23) or located near critical structures (24), with a recommended radiation dose corresponding to 20 Gy × 1, 11.6 Gy × 2, or 8.5 Gy × 3 to gain local tumor control (25). The clinical success of SRS encouraged the development of a corresponding technique to treat extracranial tumors, named SBRT or SART (26). Hence, SART is defined as a method to deliver high-dose radiation by ultra-precision external-beam RT to extracranial parts of the body using few fractions (typically 1–5) (27).

In lung cancers, high-dose RT-regimens have revolutionized treatment outcome and overall survival for early stage inoperable peripheral tumors (28–30), and are currently considered also for operable lung cancers (31). In an overview by Heinzerling et al. (28), stage I NSCLC lung tumors receiving 45–60 Gy in three fractions versus 30–34 Gy in one single-fraction demonstrated 3-year post-RT tumor control rates of 92–98% and 80%, respectively. Centrally located lung tumors, being more prone to normal tissue toxicity than peripheral tumors, may gain similar numbers of tumor control (>85%) and acceptable toxicity with a less potent dose per fraction, i.e., eight fractions of 7.5 Gy (32, 33). In addition to lung cancers, SART (15–18 Gy × 3) has also generated encouraging results in primary and secondary (34, 35) hepatic tumors (36–38) and metastatic spinal lesions (39, 40).

The successful application of high-dose RT to large brain tumors and centrally located lung tumors illustrates the concept of “risk-adapted” fractionation regimens, designed to avoid high-grade toxicity upon high-dose RT for tumors located close to critical organs (41). However, clinical data have indicated that local tumor control is not only exclusively determined by dose and fractionation but also influenced by dose-prescription (41) and tumor size (42). Accordingly, recent tumor-volume-adapted dosing-approaches demonstrated excellent local control also for SART of large-volume lung tumors (43). Additionally, by re-plotting clinical data for tumor control probability as a function of *biological effective dose* for stage I non-small cell lung cancer, it was suggested that there is no difference in tumor control for single-fraction versus multi-fraction SART (44). Important in this context is a clear distinction between novel high-dose oligo-fractionated regimens and standard protracted regimens with 25–36 fractions of 2 Gy.

In the clinics, effects of varying dose and fractionation are usually guided by the linear–quadratic (LQ)-model (45–47). This mathematical tool was developed to estimate radiation damage on normal (and neoplastic tissue), and has proved useful for designing and comparing effects of new fractionation protocols (48). The LQ-model is suggested valid up to 8–10 Gy per fraction (49), but considered inappropriate for the high-dose regimens delivered by SRS and (lung) SART (50), presumably underestimating tumor control in the high-dose range (51, 52). Therefore, based on the recent success and widespread use of ablative RT, adapted tools for comparing dose and fractionation are being discussed (53–56).

## Endothelial Cells and the Different RT-Regimens

The importance of angiogenesis and sprouting of resident endothelial cells (ECs) for continued tumor growth and metastasis has been advocated by Folkman since 1971, who also proposed the idea of killing tumor cells indirectly by targeting the ECs (57). The leaky and fragile ECs in tumors are proliferating considerably faster than ECs in normal tissues, and were early on suggested as a suitable therapeutic target (58). In the context of RT, a computer model fitted to clinical data proposed that the vascular effect contributes by 19–33% to the overall effect from single high-dose (20 Gy) radiosurgery (59). Of note, radiation effects on the tumor vasculature go beyond direct damage to the nuclear matter. Kolesnick and coworkers have long suggested that a rapid wave of ceramide-mediated apoptosis of ECs is the principal target for radiation injury and epithelial stem cell damage after high-dose RT (60–62). This group proposed a threshold dose of ~10 Gy for induction of apoptosis in tumor-associated ECs in culture (60, 63). More recently, anti-angiogenic agents (anti-VEGF or anti-VEGFR2) introduced right before high-dose RT demonstrated induction of up-regulated ceramide levels that resulted in augmented fraction of apoptotic ECs (64).

Radiation-induced changes in tumor blood vessels are known to be highly variable, depending markedly on the radiation dose and regimens [review in Ref. (13)]. Thus, during conventional (low-dose) fractionated RT, the status of the vasculature is somehow preserved, and occasionally improved or normalized (65), at least during the early part of a treatment course. These results are in line with a reported low-dose (≤5 Gy) RT stimulation of angiogenesis (66) and/or vasculogenesis (67, 68) in EC *in vitro* models. Also, in a study of radiation responses to the microcirculation of muscle flaps *in vivo*, a single dose of 8 Gy could only reduce capillary perfusion by 4.3%, implying that 8 Gy causes minimal damage to microvessels and the EC lining (69). However, at radiation doses exceeding 10 Gy/fraction, the tumor vessels become severely damaged (70) resulting in reduced blood perfusion and indirect tumor cell killing due to starvation (13). Despite such potentially, beneficial effects of high-dose RT, studies performed in animal models of intracranial glioblastoma multiforme (GBM) suggest that following local irradiation and ablation of the tumor vasculature, blood flow may be reconstituted several weeks after completed RT (71). The phenomena of such in-field recurrences are particularly common for the highly angiogenic glioblastomas, and represent a significant therapeutic challenge. Assuming that the process of angiogenesis (sprouting of vital resident ECs) is largely abrogated upon local high-dose RT (15–20 Gy), tumors rely on infiltration of circulating endothelial and other progenitor cells for revascularization and regrowth. This secondary “backup” pathway termed vasculogenesis represents a novel strategy to enhance local tumor control by RT (72, 73). Notably, even though endothelial progenitor cells (EPCs) are recognized for homing to ischemic areas where they contribute to neovascularization (74, 75), extensive research efforts has lead to the conclusion that bone-marrow-derived EPCs are contributing only marginally to (re)growth of irradiated tumors (71, 76). The source of circulating ECs (or EPCs) that are homing to irradiated tumors is therefore currently undefined (72, 77). However, it is well established that recruitment of pro-angiogenic myeloid (but not endothelial) (78) bone-marrow-derived progenitor cells (BMDCs) (71, 79) is pivotal for vascular-repair (80) and tumor regrowth, although they presumably do not incorporate into the tumor vasculature (81). So far, the role of non-bone-marrow-derived EPCs (75) in a setting of tumor relapses post-RT has been little explored (81). Intriguingly, recent studies in GBM-models point to the possibility that a fraction of tumor-associated ECs may be derived from trans-differentiation of (radioresistant) cancer stem-like cells (82–84).

Blood vessels of tumors are hyperpermeable and structurally abnormal, with a tortuous and dilated appearance and low coverage of pericytes that in total creates a microenvironment that is hypoxic and acidic, with high interstitial pressure. Hence, around 90% of all solid tumors have median oxygen levels that are lower than those appearing in normal tissues (85, 86). Tumor hypoxia is a well-known cause of radiation resistance (85), due to the fact that irradiated hypoxic cells are about 2.5–3 times less radiosensitive than normoxic cells (87). The idea that low-oxygen values contribute to radio-protect tumor cells correlates with clinical observations; severely, hypoxic tumors and high expression of hypoxia-inducible genes are generally associated with an aggressive phenotype, treatment failure, enhanced metastasis, and poor prognosis (88, 89). Considerable endeavors have been dedicated to improve radioresponses by targeting hypoxia (2, 85, 90, 91). In patients, the benefit of fractionated and protracted (low-dose) radiation regimens is partly explained by the phenomenon of tumor *re-oxygenation*, where surviving hypoxic cells become oxygenated between fractions (92). Conversely, a consequence of single high-dose RT is disrupted vasculature and invalidated angiogenesis, which may lead to a large increase in tumor hypoxia (71). Currently, there is an ongoing debate among scholars whether the dose-boosting offered by oligo-fractionated SART is sufficient to counterbalance the loss/reduced contribution of re-oxygenation (93) or if SART should be combined with a hypoxic radiosensitizer (42, 94). The latter alternative offers the possibility of achieving similar tumor control rates with a reduced radiation dose, and hence reduced toxicity, in patients (42, 90).

Prevention of revascularization post-radiation represents an appealing strategy to radio-sensitize tumors. Restoration of (radiation-) damaged vasculature by colonizing progenitor cells is initiated by HIF-1 (hypoxia-inducible factor-1) (95), the HIF-1-dependent and -independent (67) expression of SDF-1 (stromal cell-derived factor-1) (96) and the potent angiogenesis regulator VEGF (vascular endothelial growth factor). In a preclinical study with local (whole brain) irradiation of GBM-xenografts using a single high-dose (15 Gy), perfusion was reduced to a minimum after 2 weeks, whereas hypoxia, HIF-1 and SDF-1 was maximal at the same time point (71). SDF-1, secreted mainly by reactive tumor-associated fibroblasts (97), is retained in hypoxic tissues where it binds to the receptors CXCR-4 (96) and CXCR-7 expressed on monocytes and ECs (72), respectively. Recent studies indicate that hypoxia increases the expression of CXCR-7 in the pulmonary endothelium (98). Moreover, CXCR-7 is a scavenger receptor (99) responsible for uptake and degradation of SDF-1 (100) as well as mediating EC regeneration, repair, and proliferation (98, 101). Interestingly, inhibitors of CXCR-4, CXCR-7, and SDF-1 have all demonstrated delayed (or blocked) tumor recurrence post-RT in animal tumor growth-delay experiments (72). Regarding regulation of SDF-1 expression by RT, some opposite observations have been reported. Whereas secreted levels of SDF-1 from cultured lung (NSCLC)-CAFs are significantly reduced after 18 Gy × 1 (14), local high-dose irradiation to mice xenografts of GBM, breast, and lung tumors followed by detection in tissue-sections has indicated increased intratumoral SDF-1 quantity already 2 days post-RT (78), with maximum levels appearing after 14 days (71). In the latter system, the vasculogenesis-pathway could be blocked by interfering with the SDF-1/CXCR-4 interactions, and resulted in tumor control at radiation doses that alone were insufficient for sterilizing the tumors (71). The HIF-1 target-gene VEGF is another important factor required for homing and retention of circulating mononuclear myeloid cells (80). However, inhibition of VEGF was demonstrated to be less efficient than inhibition of the SDF-1/CXCR-4 interactions in abrogating tumor reperfusion and regrowth post-RT (71). Interestingly, in the same GBM-model, the radiation-induced influx of BMDCs was demonstrated to be dose-dependent, with doubled levels appearing after local (whole brain) irradiation with 15 versus 8 Gy (71). Nevertheless, when combining inhibition of SDF-1/CXCR-4 interactions with radiation, a fractionated low-dose regimen (2 Gy × 5) and a single high-dose (15 Gy) both resulted in complete anti-tumor responses (71).

## Immune Cells and the Different RT-Regimens

Immune escape is the process in which malignant cells become unrecognized by the host immune system. The steady integration of multiple immune escape mechanisms by tumors during its progressive growth is now acknowledged as one of the hallmarks in cancer (6). During tumor development, not only malignant cells but also many other tumor-infiltrating cells undergo a process of immune editing, developing mechanisms by which the tumor tissue escapes immune recognition and elimination. Similar to angiogenesis, IR may act as a double-edged sword on inflammation and the immune system. Originally, RT was considered to be immuno-suppressive rather than immuno-stimulatory, largely due to the procedure of whole-body-radiation to minimize the immune-responses connected with bone-marrow transplantation. Also, it has been clinically documented that low-dose X-ray irradiation exerts an anti-inflammatory effect on benign diseases and chronic degenerative disorders (102). Additionally, immune-suppressive effects may come from the collateral damage exerted on tissue-resident antigen-presenting cells. On the other hand, irradiation may induce inflammation and immune reactions by the generation of tumor antigens, oxygen radicals, and alarm signals (8). Interestingly, an abscopal off-target response to RT has occasionally been reported in metastatic cancer patients, denoting the phenomenon of remote tumor regression outside the irradiated field after local RT (103, 104). The renewed interest of using RT as an immune adjuvant has already stimulated scientific efforts to decipher the mechanisms behind radiation-induced immune-responses and has generated novel concepts as “immunogenic cell death” to explain these events (105). In the following text, we present some of the important immunogenic pathways regulated by RT and the impact RT may exert on the different cellular components of the immune system.

One of the principal mechanisms for immune escape of tumor cells is the reduction of MHC-I levels on their plasma membrane. Notably, RT has been shown to upregulate MHC-I expression (106, 107). Moreover, MHC-I up-regulation is dose and time dependent, having a significant effect at doses above 4 Gy in melanoma cell-lines and at 8–20 Gy in murine colon carcinoma MC38 cells (108, 109). A potential explanation to this observation may be found in the induction of IFN-γ in the tumor microenvironment mainly by infiltrating T-lymphocytes and NK cells (110). RT is also able to modulate other surface molecules with immune modulating functions. ICAM-1 induction by RT has been observed in an array of different human and murine cell-lines, but mainly after high-dose radiation (111). On the other hand, radiation-induced up-regulation of the death-receptor CD95 is observed in various tumor types after different doses of radiation (112). The prominent role of RT on modulating phenotypic markers on neoplastic cells significant for their immunogenicity is reflected by the clear synergistic effect between irradiation and immunotherapeutic approaches using either specific blockage of immune-regulatory receptors (113), intratumoral administration of immune-stimulatory cytokines (114), or adoptive transfer of tumor-specific CTLs (115).

Damage-associated molecular patterns (DAMPs), or danger signals, are an important part for initiation of effective immune activation. DAMPs are small molecules released by either tumor cells or the miscellaneous inflammatory cells present in the tumor stroma. These molecules are generally released in response to stress situations as the ones created by RT. Some of the best characterized are high motility group protein 1 (HMGB1), calreticulin (CRT), heat shock proteins (HSPs) 70 and 90, adenosine triphosphate (ATP) and uric acid (105). Radiation can induce different cell death pathways depending on multiple factors such as fraction numbers and doses, tumor-type, tumor stage, and microenvironment. Tumor cell apoptosis is triggered most likely at low doses of radiation. Apoptotic cell death shows patterns of both tolerogenic and immunogenic cell death, viewed as CD47 up-regulation and oxidized HMGB1 release, and CRT externalization and reduced HMGB1 release, respectively. Radiation at high-doses on the other hand promotes necrotic cell death (116, 117). During necrosis, the cell membrane becomes disintegrated, which lead to an uncontrolled release of the cytoplasmic content into the extracellular space; this could include DAMPs and pro-inflammatory cytokines. Necrotic cell death is thus considered immunogenic but only if the process is accompanied by the release of stress signals. Radiation can also modulate the expression of certain chemokines and cytokines released by both tumor cells and stromal cells (8, 118). However, each single molecule can be both stimulatory and inhibitory depending on the target cells, concentrations, microenvironmental conditions, as well as tumor stage and type. Importantly, many of these factors are directly responsible for the systemic effects of local irradiation. Among others, factors such as IFN-γ, GM-CSF, TNF-α, IL-6, IL-10, or TGF-β account as powerful immune-modulating molecules potentially modified by IR with properties that can mediate systemic effects (118–121). Of note, the origin of these cytokines is not restricted to tumor cells, as also host cells from the tumor microenvironment constitute an important source (14, 122, 123).

Macrophage (Mφ) infiltration into tumors is a frequently observed phenomenon, usually accumulated at the border of malignant tissues and close to necrotic areas, with a highly variable abundance that depends on tumor-type and stage (124, 125). The role of Mφ in anti-tumor immunity is reliant on their phenotype, type-1 being considered immune-stimulating and type-2 immune-suppressive. Previous reports have suggested that local irradiation favors the Mφ-2 phenotype with tolerogenic functions, most likely due to RT-induction of immune inhibitory molecules such as COX-2/PGE2 and NO (126). This behavior seems to be associated with both single high-dose (20 Gy) and fractionated regimens (4 Gy × 15). Moreover, some groups have observed that tumor-associated Mø (TAM) in high numbers correlates with increased radio-resistance of tumors (127). Likewise, others have demonstrated that selective elimination of TAMs increases radiosensitivity in B16 murine melanomas *in vivo* (128). Interestingly, recent reports are indicating that low-dose irradiation redirects macrophage differentiation from the immune-suppressive state (M2) to one that enables recruitment of cytotoxic T-cells (65) (M1). CD8^+^ T-cell recruitment and expansion in tumors is a good prognostic factor in many tumor types, whereas intratumoral Tregs are important promoters and stabilizers of immuno-suppressive conditions, therefore associated with poor prognosis (129). Preclinical studies on radiation-mediated effects on lymphocyte levels and profiles are somewhat contradictory, with some reports showing positive effects and others showing the opposite (130, 131). Most likely, the conflicting literature available underlines the fact that the immunological profile of cancers is tumor-type specific, and that local microenvironmental parameters such as degree of hypoxia, intratumoral pH, tumor stroma composition, and cytokine milieu are indeed key factors influencing local and systemic immune-responses after RT. Dendritic cells are also key elements for proper initiation of adaptive immune reactions, but are frequently present at low numbers within the tumor tissues, and very often the infiltrating DC is plasmacytoid DC or immature DC with a clear immuno-suppressive phenotype (132). Immunotherapy aimed at reversing the immuno-suppressive status of DC contemplates the administration of cytokines that stimulate DC maturation and/or numbers, or the local injection of exogenously activated autologous DC (133). Radiation-induced DAMPs during cell death and the enhanced amounts of tumor antigens released may exert an impact on DC activation; thus, following the aforementioned explanations, different radiation doses and regimens may influence differently the activation of DC.

Apposite stimulation of the immune system by RT is probably the most illustrative example of indirect mechanisms contributing to tumor control, working in parallel to the more widely perceived idea of DNA damage in neoplastic tumor cells. Some of the first issues that need clarification for optimal induction of immune-responses are related to radiation dose and fractionation (103). Several authors claim that high-dose RT mounts a stronger immune attack against tumors than low-dose RT. This effect is supported by activation of different immunogenic mechanisms such as (a) secretion of higher levels of alarmin protein HMGB1 by dying tumor cells (10 Gy × 1) (117); (b) increased intratumoral production of type-I IFN by tumor-associated DCs (25 Gy × 1) (134); (c) increased number of CD45^+^ cells infiltrating tumors (15 Gy × 1), and a higher number of lymphocytes expressing IFN-γ (131); (d) increased number of tumor-specific CD8^+^ T-cells supported by the activation of tumor-infiltrating DCs (10 Gy × 1) (135); or (e) inducing more efficient maturation of antigen-presenting DCs (135) with enhanced cell-surface expression of MHC-I (10 Gy × 1) (108). Notably, two reports have suggested that RT-induced immune-responses depend on (oligo)fractionated rather than single-dose regimens (113, 136). Local irradiation of B16-OVA melanoma in mice revealed that a single (medium-high) dose of 7.5 Gy and above, but not 5 Gy, generated tumor immunity (136). An intriguing observation in that study was the fact that at the highest dose tested (15 Gy × 1), the authors reported intratumoral accumulation of T-regulatory cells, a reaction that could hinder the adaptive tumor immunity exerted by RT. In line with the last observation, the immuno-suppressive cytokine TGF-β (137) in growth medium of irradiated fibroblasts demonstrated base-line levels after 5 Gy, but significantly increased levels after 15 and 20 Gy (138). In the study by Dewan et al. (113), an abscopal effect was observed only when immunotherapy (anti-CTLA-4 antibodies) was combined with (3–5) fractions of medium–high-dose RT but not with single-high-dose (20 Gy × 1). The introduction of such medium–high radiation doses in the clinics has resulted in corresponding novel oligo-fractionated regimens that clearly contrasts the classical protracted 2 Gy-regimens typically utilized in curative settings for the last 80 years.

## Fibroblasts and the Different RT-Regimens

Much of our acquired knowledge on fibroblast responses to IR emerge from studies aimed at understanding normal tissue reactions to RT, using normal connective tissue fibroblasts as experimental models. Genome-wide studies have tried to decode the overall changes in gene-expression on normal tissue fibroblasts exposed to IR (139–141). After functional categorization of differentially expressed genes, those reports clearly reveal that some central cellular pathways become persistently altered by radiation including DNA-damage responses, regulation of cell-cycle and proliferation, programed cell death, p53 target-genes, and some growth factor receptor signaling pathways (142). Similar responses have been described also for lung-CAFs (143). From a clinical point-of-view, parameters such as fraction-size and number of fractions seem to play an important role on fibroblast responses to IR. Hence, studies on transcriptome alterations induced by IR on fibroblasts have revealed marked differences at escalating radiation doses (140). In another study, ROS scavenging pathways and ECM remodeling pathways were more potently altered when fibroblasts were challenged with larger total dose and fractionated regimens than with single low doses (139).

A common observation made by many laboratories is that the fibroblast is a rather radioresistant cell-type, surviving single doses above 50 Gy in cell culture (140, 144–146). However, when the radiation dose exceeds 10 Gy, the cellular phenotype becomes profoundly altered, characterized by induction of irreversible DNA-damage response and concomitant development of stress-induced cellular senescence (146, 147). Senescent fibroblasts are by definition trapped in a state of permanent growth-arrest, but are still metabolically active and may therefore influence tissue responses to damage. In fact, numerous reports have highlighted the strong influence of senescent fibroblasts in the regulation of inflammation and the growth of adjacent epithelial cells (148). In a cancer setting, senescent fibroblasts are considered to play deleterious roles by contributing to sustained chronic inflammatory reactions, promoting angiogenesis and nourishing growth and invasion of neoplastic cells. These pro-malignant events are primarily driven by the release of soluble paracrine factors globally referred to as the senescence-associated secretory phenotype or SASP (149, 150). The SASP involves secretion of numerous inflammatory mediators, extracellular matrix proteins, proteases, and growth factors that collectively can render the microenvironment favorable to tumor growth. However, some authors argue in the opposite direction. Studies performed on tissue specimens from the post-irradiated breast indicate that malignant cells may be observed in irradiated tissues, while tumors often remain in a dormant state (151). A potential explanation to this observation may rely not only on the intrinsic susceptibility of cancer cells to the therapy but also on changes in the supportive stroma that makes the environment non-permissible for tumor regrowth (152).

## Cancer-Associated Fibroblasts and the Different RT-Regimens

Collectively, most previous literature highlights the pro-malignant phenotype acquired by fibroblasts after radiation exposure. However, it should be noticed that the large majority of these studies have been conducted with normal tissue fibroblasts and/or fibroblast cell-lines, but not with resident and activated tumor-associated fibroblasts. To understand the contribution of CAFs to therapeutic outcomes after RT, it is utterly important to take into consideration the activated nature of tumor-resident fibroblasts (123, 153, 154). Non-irradiated reactive lung-CAFs actively produce numerous tumor-promoting molecules such as matrix metalloproteases, inflammatory cytokines, pro-angiogenic factors, and other growth factors (14). Also non-irradiated (activated) CAFs from skin, breast, and pancreatic cancer possess a pro-inflammatory signature, including up-regulated levels of IL-6 (155), SDF-1 (97), and TGF-β (156). Lung-CAFs exposed to ablative RT (18 Gy) demonstrated reduced expression of pro-angiogenic molecules such as SDF-1, angiogenin, and angiopoietin-1, and a concomitant reduction in migration-rates of ECs. In the context of high-dose RT, while human diploid fibroblasts demonstrated enhanced levels of IL-6 (147), primary normal fibroblasts (138) or lung-CAFs did not show altered expression of this cytokine. Regarding the growth factors HGF and bFGF, two separate reports have shown unchanged levels of the first and enhanced levels of the second, after high-dose RT in lung-CAFs (14) and PCC-CAFs (157). Activation of HGF-receptor is suggested to contribute to the enhanced invasiveness of PCCs grown in co-culture with CAFs (157). In xenograft models, senescent fibroblasts co-transplanted with cancer cells were found to increase tumorigenic effects on cancer cells (145, 158). However, the overall tumor regulatory properties of senescent fibroblasts remain controversial. It is still unresolved if this phenomenon is tumor-type specific or organ specific, or whether the senescent phenotype acquired after high-dose radiation exposure is comparable to other forms of senescence. In this regard, some authors have suggested that cancer promotion by senescent stromal cells may be restricted to certain organs and tissue types and claim that the importance of senescent cells needs to be validated in other tissues than subcutaneously grown tumors (159). All in all, existing literature on the contribution of irradiated fibroblasts to cancer relapse or eradication remains puzzling. Dose and fractionation regimens may play a central role on the ultimate response of CAFs to treatment. Additionally, important aspects such as the fibroblast-mediated immune-regulatory effects after irradiation still need to be explored.

## Conclusions and Future Perspectives

Today it is widely acknowledged that the non-malignant components of solid neoplasms play central roles in the overall responses of cancers to therapies. Radiation therapy is a unique therapeutic modality that targets a defined tumor-volume, which implies that all cell types within the radiation field receive the prescribed radiation dose. This insult provokes the activation of cell death mechanisms and cell damage programs that greatly influence the therapeutic outcomes. Accordingly, it becomes a priority task for the scientific community to uncover all potential side signals and events occurring during and after the course of radiation in non-malignant tumor components that could influence tumor evolution and even the fate of distant metastasis.

Stereotactic ablative RT has recently emerged as a therapeutic alternative that is already proving its efficacy for treating cancers with very poor prognosis. The hype created around this therapeutic modality is fostering the interest of scientists to investigate on the different radiobiological responses elicited on different tumor constituents by different radiation regimens. In the context of RT in general, it is utterly important to consider the different scenarios represented by variables such as dose-per-fraction, number of fractions, total dose, and overall treatment time. Thus, conventional protracted regimens in the clinics comprise multiple doses (20–36) given at low doses (~2 Gy). On the other hand, SART-regimens are meant to be given in few fractions (1–5) and higher doses (5–30 Gy). However, *in vivo* and *in vitro* radiobiological studies have revealed substantially different responses triggered by different regimens. For instance, important variations are observed between single high- or oligo high-doses, both regimens within the frame of SART. Also, medium–high-doses (5–10 Gy) may exert critically different responses than high-doses (>10 Gy). All these observations should alert researchers and practitioners at the time of choosing radiation regimens that are meant to give optimal outcomes.

Not only malignant cells, but also tumor-infiltrating host cells respond differently to different radiation regimens. ECs and the tumor vasculature seem to be preserved at the low doses used in conventional RT-regimens. On the contrary, damages to tumor blood vessels are mild at doses ranging 5–10 Gy, and severe at doses above 10 Gy. On the consequences brought by damages inflicted to the vasculature by RT, still two confronted arguments remain: does the preservation and/or normalization of tumor blood vessels in conventional protracted regimens permit a better oxygenation of tumor cells and thus provide higher radiosensitivity, or will severe ablation of tumor vasculature by SART aid to a more efficient tumor cell killing by starvation? The rate of EC damage and the extent of hypoxia are two important and interconnected factors that add certain degree of complexity to predict beneficial or detrimental effects elicited by SART. With the recent enthusiasm and extended clinical application of high-dose RT in curative settings, exemplified by SART for lung cancers, these previous results are gaining clinical relevance.

Tumor-associated fibroblasts are able to repair DNA damages induced by low radiation doses; however, at doses above 10–12 Gy, the DNA-damage response becomes permanent and the cells enter in a permanent stage of senescence. Senescent fibroblasts are metabolically active and may secrete soluble signals and enzymes that in turn could regulate the growth and behavior of neighboring cancer cells. It remains to be elucidated if irradiated fibroblasts or CAFs certainly contribute to tumor evolution post-RT, and if so which molecules and pathways are implicated in this regulation. Also, it remains uncertain if normal tissue fibroblasts and CAFs respond in the same way to RT, or if medium–high-doses (8–10 Gy) exert different effects than higher doses (15–20 Gy) in these cells.

The notion of using RT for boosting anti-tumor immunity is not new; however, at present we know that conventional RT is unable to mount efficient anti-tumor immune-responses as a single treatment modality. In the new era of SART, the interest on using radiation as an efficacious immune-stimulant is being renewed. Evidences in this field are demonstrating that high-dose regimens are more efficient to trigger both innate and adaptive anti-tumor immunity than low-dose regimens. Expression of alarm signals, induced intratumoral levels of interferons, efficient maturation of DCs, and controlled immune-regulatory mechanisms count as some of the important mechanisms behind the effect. Importantly, some reports argue that oligo-fractionated regimens work better than single-high-dose regimens, and also that medium–high (7–10 Gy) doses may work better than very high doses (>15 Gy). It is now most widely assumed that radiation probably can only amplify or augment the pro-immunogenic status in tumors but will hardly be able to change by itself a well-settled immuno-suppressive environment into an immune-stimulating one. Therefore, current research is now focused on the successful combination of immune modulating therapies and the optimal RT protocols.

Different cellular components are found in tumors, and as such, different responses are taking place at the same time incited by RT. Only if we globally consider all events, in a system biology way of thinking, could we predict more precisely the ultimate consequences of the therapy. Dissecting the responses undergone by the different constituents of the tumor stroma under different RT-regimens is aiding us to more efficiently choose the adequate treatment schedules in RT. Finally, concepts such as stereotactic radiation or high-dose radiation are getting too broad and may lead to undesired misinterpretations. Given the striking different responses of cells to small variations in the schedules, a more precise terminology would be needed among professionals to define radiation regimens and treatment schedules.

## Conflict of Interest Statement

The authors declare that the research was conducted in the absence of any commercial or financial relationships that could be construed as a potential conflict of interest.
